# The selenium-containing drug ebselen potently disrupts LEDGF/p75-HIV-1 integrase interaction by targeting LEDGF/p75

**DOI:** 10.1080/14756366.2020.1743282

**Published:** 2020-03-31

**Authors:** Da-Wei Zhang, Hao-Li Yan, Xiao-Shuang Xu, Lei Xu, Zhi-Hui Yin, Shan Chang, Heng Luo

**Affiliations:** a Institute of Bioinformatics and Medical Engineering, School of Electrical and Information Engineering, Jiangsu University of Technology, Changzhou, China; b Center for Food and Drug Evaluation & Inspection of Henan, Zhengzhou, China; c First Hospital of Shanxi Medical University, Taiyuan, China; d College of Life Sciences, South-Central University for Nationalities, Wuhan, China

**Keywords:** ALLNIs, integrase, LEDGF/p75-integrase interaction, HIV-1

## Abstract

Lens-epithelium-derived growth-factor (LEDGF/p75)-binding site on HIV-1 integrase (IN), is an attractive target for antiviral chemotherapy. Small-molecule compounds binding to this site are referred as LEDGF-IN inhibitors (LEDGINs). In this study, compound libraries were screened to identify new inhibitors of LEDGF/p75-IN interaction. Ebselen (2-phenyl-1,2-benzisoselenazol-3-one), a reported anti-HIV-1 agent, was identified as a moderate micromolar inhibitor of LEDGF/p75-IN interaction. Ebselen inhibited the interaction by binding to LEDGF/p75 and the ability of ebselen to inhibit the interaction could be reversed by dithiothreitol (DTT). BLI experiment showed that ebselen probably formed selenium-sulphur bonds with reduced thiols in LEDGF/p75. To the best of our knowledge, we showed for the first time that small-molecule compound, ebselen inhibited LEDGF/p75-IN interaction by directly binding to LEDGF/p75. The compound discovered here could be used as probe compounds to design and develop new disrupter of LEDGF/p75-IN interaction.

## Introduction

HIV-1, the virus that causes acquired immune deficiency syndrome (AIDS), is one of the world’s most serious health and development challenges. There were approximately 36.7 million people worldwide living with HIV/AIDS at the end of 2016^1^. During the last three decades, significant progress has been made in the medical treatment of patients with HIV-1 infection; however, the rapid emergence of drug resistance together with toxicity and patient compliance limit the use of antiviral drugs, there remains a need for discovery of new antiviral agents^2^^,^^3^. HIV-1 integrase (IN), an essential enzyme encoded at the 3′-end of the HIV pol gene, is an attractive target for chemotherapeutic intervention^4^^,^^5^. IN is a multifaceted player in HIV-1 infection. Apart from its catalytic activity composed of 3′ processing and strand transfer, investigation of mutagenesis in IN and the mode of action of allosteric IN inhibitor (ALLINI) revealed that IN also play several other biological roles in HIV-1 life cycle, including virion morphogenesis, virus particle uncoating and PIC nuclear import^6^.

Currently, there are four approved HIV-1 IN inhibitors, including raltegravir, elvitegravir, dolutegravir and bictegravir, which block the strand-transfer step by binding to the active site in IN and designated as integrase-strand-transfer inhibitor (INSTI)^7–11^. During the last decades, ALLINI has emerged as a promising and complementary approach to the use of INSTI. ALLINIs, alternatively referred as lens-epithelium-derived growth-factor (LEDGF/p75)-integrase inhibitors (LEDGINs), noncatalytic site integrase inhibitors (NCINIs), IN-LEDGF/p75 allosteric inhibitors (INLAIs) and selective multimeric IN inhibitors (MINIs), are mechanistically distinct from active-site inhibitors INSTIs and therefore provide an important clinical complement to INSTIs in the clinical treatment of HIV-1 infection^12–18^.

**Figure 1.**
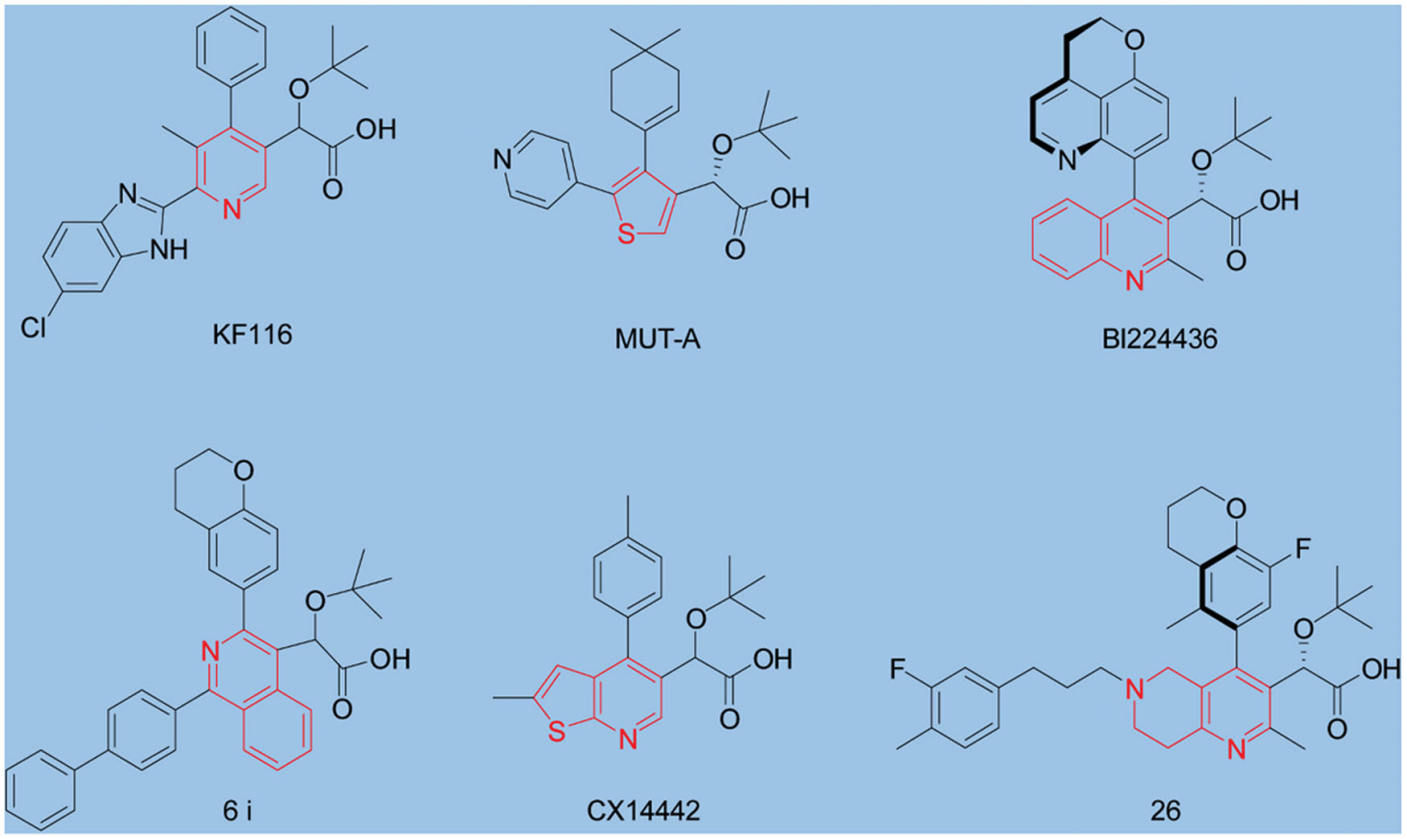
Structures of representative ALLINIs. Chemotypes of each ALLNI are highlighted in red colour.

Lens epithelium-derived growth factor (LEDGF/p75) is a cellular cofactor hijacked by HIV-1 virus to tethers the integration complex to chromatin, thereby facilitating viral integration into host-cell DNA^19^. LEDGF interacts with HIV integrase (IN) through its C-terminal integrase binding domain (IBD, amino-acid residues 347–429)^20^. Disruption of the LEDGF/p75-integrase protein-protein can efficiently block HIV-1 replication^21^. During the last decade, several small molecules (representative chemotypes showed in Figure 1) have been identified as disruptors of the LEDGF/p75-HIV-1 IN interaction, which bind to the IN catalytic core domain (CCD) dimer interface in the LEDGF/p75 binding pocket^6^. Although these agents (ALLINIs) do indeed bind to the LEDGF/p75 interface on IN *in vitro*, their primary mechanism of action instead is to block viral-particle maturation during the late stages of viral replication^16,^^22–24^. And further multiple studies have elucidated the late stage mechanism of ALLINIs: ALLINIs stimulate aberrant or hyper-multimerization of IN thus impairing IN binding to vRNA required for particle morphogenesis and causing the mislocalization of ribonucleoprotein complex (RNPs) outside of the conical core made of capsid proteins^25^^,^^26^.

Recently discovered ALLINIs all inhibit HIV-1 replication by occupying the LEDGF/p75 pocket in IN. Although, cyclic peptides targeting LEDF were discovered to inhibit the LEDGF/p75–IN interaction^27^^,^^28^, there are no potent small-molecule inhibitors directly binding to the LEDGF/p75 to date. In this study, we used an HTRF-based assay to identify novel disrupters of LEDGF/p75-IN interaction by screening compound libraries. In a previous study, ebselen was reported as HIV-1 capsid inhibitor, which inhibits the replication of HIV-1 with a 50% effective concentration (EC_50_) of 1.99 µM and has a half-maximal cytotoxic concentration (CC_50_) of 25.4 µM. In present study, ebselen was identified as ligand binding to the LEDGF/p75 and inhibitor of LEDGF/p75-IN interaction. This work provides proof-of-concept for direct targeting of LEDGF/p75 by small molecule as novel therapeutic strategy and the compound here serve as leads for future development of new inhibitors of LEDGF/p75-IN interaction *in vitro*.

## Material and methods

### Agents and inhibitor libraries

All general biochemical reagents were obtained from AMRESCO (Solon, USA). Ni-NTA resin and GST resin were purchased from Smart-Lifesciences (Changzhou, China). Anti-6His-XL665- and anti-GST-Eu Cryptate antibodies were purchased from Cisbio. 96-well Black microplates were purchased from Greiner Bio-One. White 384-shallow well microplate was purchased from PerkinElmer. Streptavidin (SA) biosensors were purchased from Pall ForteBio. EZ-Link™ Sulfo-NHS-LC-LC-Biotin were purchased from Thermo Fisher Scientific. Ebselen was purchased from MedChem Express (Shanghai, China). Three protein kinase inhibitor libraries (Syn kinase inhibitor library, protein kinase inhibitor library and kinase inhibitor library) and REDOX library were obtained from National Compound Resource Center (Shanghai, China).

### *In vitro* IBD-IN interaction assay

Recombinant IBD with *N*-terminal glutathione S-transferase (GST-IBD) and IN with *N*-terminal hexahistidine tag (His6-HIV-1 IN) were expressed and purified as previously described^29^. An assay based on a homogeneous time-resolved fluorescence resonance energy transfer (HTRF) was used to measure the interaction between HIV-1 IN and IBD according to a previous study^30^. The assay was performed in white 384-shallow well microplate. Prepare “Protein MIX” by mixing His6-HIV-1 IN (final concentration of 50 nM) and GST-IBD (final concentration of 25) in the assay buffer (25-mM Tris-HCl pH 7.5, 150-mM NaCl, 1 mg/ml BSA, 0.1% NP40, 2-mM MgCl_2_). 8 µl “Protein Mix” and 2 µl assay buffer were added to the plate and incubated at 25 °C for 30 min, followed by the addition of 10 µl of premixed antibodies (5 µµl anti-His XL665 [4 nM] and 5 µl anti-GST europium cryptate [0.8 nM]) in the assay buffer with 100-mM KF. The plate was incubated in the dark for 1 h at 25 °C. Finally, the plate was read in an Envision 2102 multilabel reader (PerkinElmer Life Sciences). Raw counts (in counts/s) at 665 and 620 nm were collected, and the signal was calculated as the ratio of (cps 665:620 nm) × 10,000.

### Screening assay

Add 8-μL protein MIX to each well in a white 384-shallow well microplate. To the 8 μL protein MIX, add and mix thoroughly 2 μL of DMSO or compound (50 µM) dissolved in DMSO. The concentrations of DMSO in the assay should be no more than 4%. Incubate the plate for 30 min at 25 °C. Add 10 μL premixed antibodies and mix. Incubate the assay plate in the dark for 1 h at 25 °C. Perform the HTRF measurement in an Envision 2102 multilabel reader. The Z’ factor were calculated to evaluate the screening results of each plate^31^. Data were analysed and visualised in GraphPad Prism 5.0.

### Dose-response curves

Percentages of inhibition at different concentrations were determined using the same reaction conditions as primary screen. All measurements were performed as 12-point (the range of compound concentration from 0.024 to 50 µM) dose–response curves. BI 224436 was used as a reference inhibitor. Data analysis was performed and visualised using GraphPad Prism 5.0 nonlinear curve fitting.

### Biolayer interferometry assay

A protein binding assay was performed by biolayer interferometry (BLI) as described previously^32^. First, purified recombinant LEDGF/p75 protein was biotinylated using the Thermo EZLink long-chain biotinylation reagent. Then, biolayer interferometry (BLI) assay was performed using an OctetRED96 instrument from PALL/ForteBio. All assays were run at 30 °C with continuous 1000 rpm shaking. PBS with 0.01% Tween-20 was used as the assay buffer. Briefly, biotinylated LEDGF/p75 protein was tethered on Super Streptavidin (SSA) biosensors (ForteBio) by dipping sensors into 200 µl per well 50 µg/ml protein solutions. The measurement processes were all under computer control. Programme procedures were established as follows: For the initial step, biosensors were washed in assay buffer for 300 s to form a baseline; the biosensors labelled with biotin-LEDGF/p75 were exposed to 100 µM compounds for association, and were monitored for 600 s; and then, the biosensors were moved back into assay buffer to disassociate for another 1800s. Data were fit globally and generated automatically by Octet User software (version 9.0.0.10; Fortebio).

## Results and discussion

**Figure 2.**
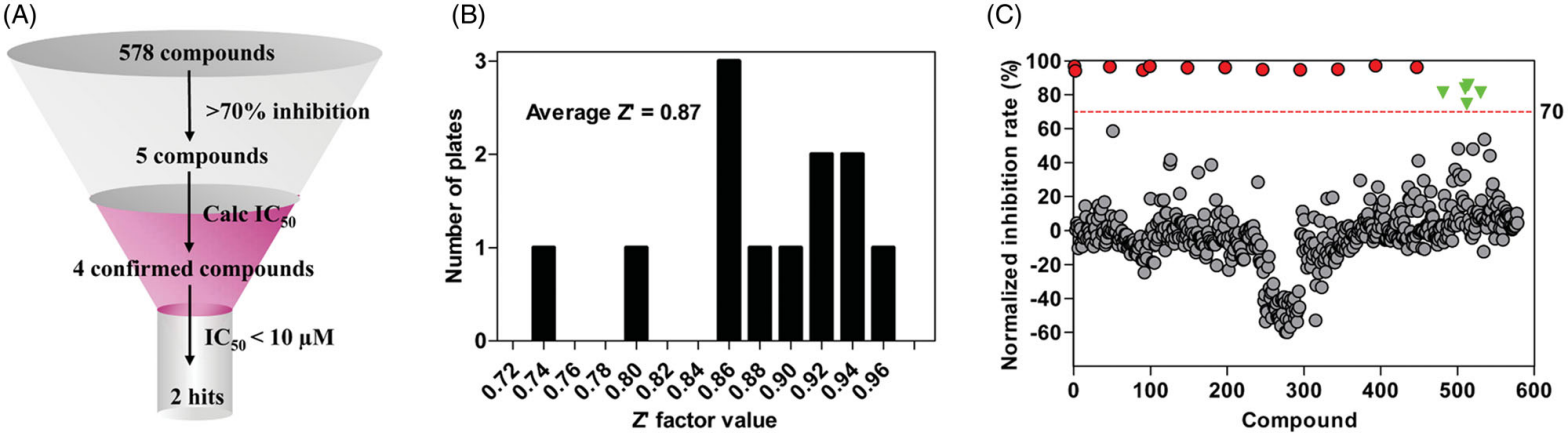
Overview and primary screening results. (A) Screening cascade. (B) Z-factor frequency distribution for 12 screening plates. (C) Replicate plot from screening 578 compounds for disruption of LEDGF/p75 IBD-IN interaction at 50 µM. The red dash line indicates our cut-off point of 70% inhibition and 5 compounds inhibited the interaction by more than 70%.

**Figure 3.**
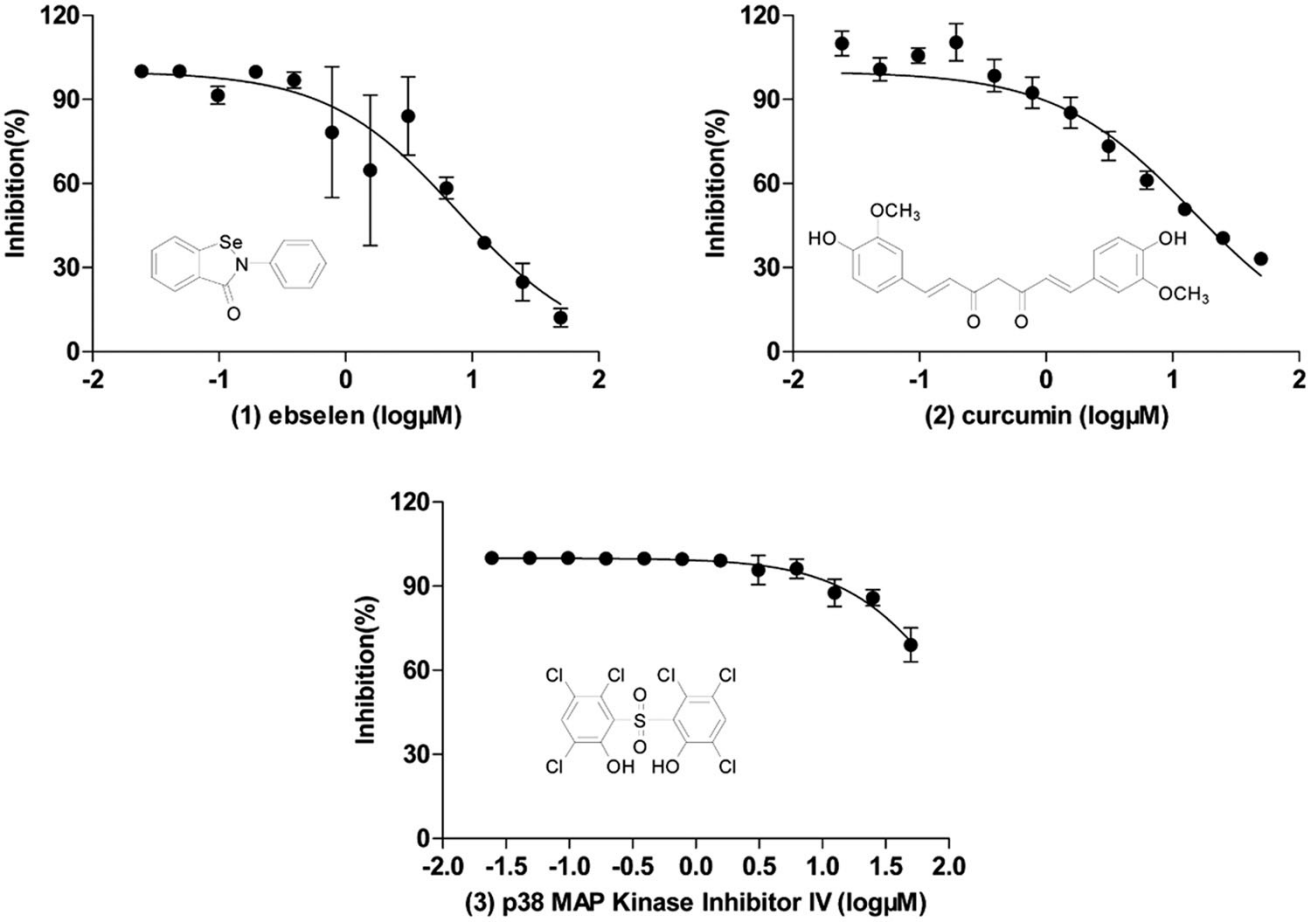
Structures and dose–response curves of confirmed positives 1–3. Data represent the mean ± SD of three independent experiments.

The IBD was previously shown to be necessary and sufficient for the interaction with HIV-1 IN^33^. In this study, the interaction between IBD (truncated form of LEDGF/p75) and IN was used to screen inhibitors of LEDGF/p75-IN interaction. To discover new chemicals disrupting LEDGF/p75-HIV-1 IN interaction, we screened at 50 µM four libraries (Syn kinase inhibitor library, protein kinase inhibitor library, kinase inhibitor library and REDOX library) of 578 compounds. Overview of the screening process is summarised in Figure 2(A). The average Z’ factor value for assays is 0.61 and no plates failed during screening (Figure 2(B)). Out of these compounds, 5 molecules displayed a percentage of inhibition above threshold (70%) at the screening concentration (Figure 2(C)). Dose–response curves were performed and 3 compounds (1–3) were confirmed (0.17% hit rate), with IC_50_s ranging from 7.70 µM to 116 µM: curcumin, p38 MAP Kinase Inhibitor IV and ebselen, which display unique structural motifs and activities (Figure 3 and Table 1). This hit rate is comparable to those published with other drug libraries (NIH Clinical Collections libraries, 2P2I-Oriented Chemical Library……) screened on isolated targets (Table 2). Out of these compounds, we selected ebselen (1), for further investigation of mode of action and/or binding.

**Table 1.** Reported hit rates for the screening of libraries drugs

Target	Acronym	Hit rate (%)	Screened library (size)		No. of hits	**Reference**
Insulin-degrading enzyme	IDE	0.45	APTEEUS-Universite de Lille Library	1120	5	Leroux et al.^42^
Human 3-hydroxy-3-methyl-glutaryl-coenzyme A	HMG-CoA reductase	3.3	NIH Clinical Collections librarie	727	24	Bessoff et al.^43^
Aspergillus fumigatus chitinase A1	AfChiA1	0.08	Dundee Drug Discovery Unit diversity	59904	48	Lockhart et al.^44^
Dengue virus non-structural proteins NS3 and NS interaction	NS3/NS5	1.6	2P2I-Oriented Chemical Library	1664	26	Milhas et al.^45^
HIV virus Nef and Hck kinase interaction	Nef/SH3-Hck	0.2	2P2I-Oriented Chemical Library	1664	2	Milhas et al.^45^
HIV virus integrase and LEDGF/p75 IBD domain	IN/IBD	0.17	Protein kinase inhibitor library, REDOX library	578	1	This paper

**Table 2.** Inhibitory potencies of confirmed positives

Compound	Name	IC_50_ (µM)
1	Ebselen	7.70
2	Curcumin	14.52
3	p38 MAP kinase inhibitor IV	116.90

**Figure 4.**
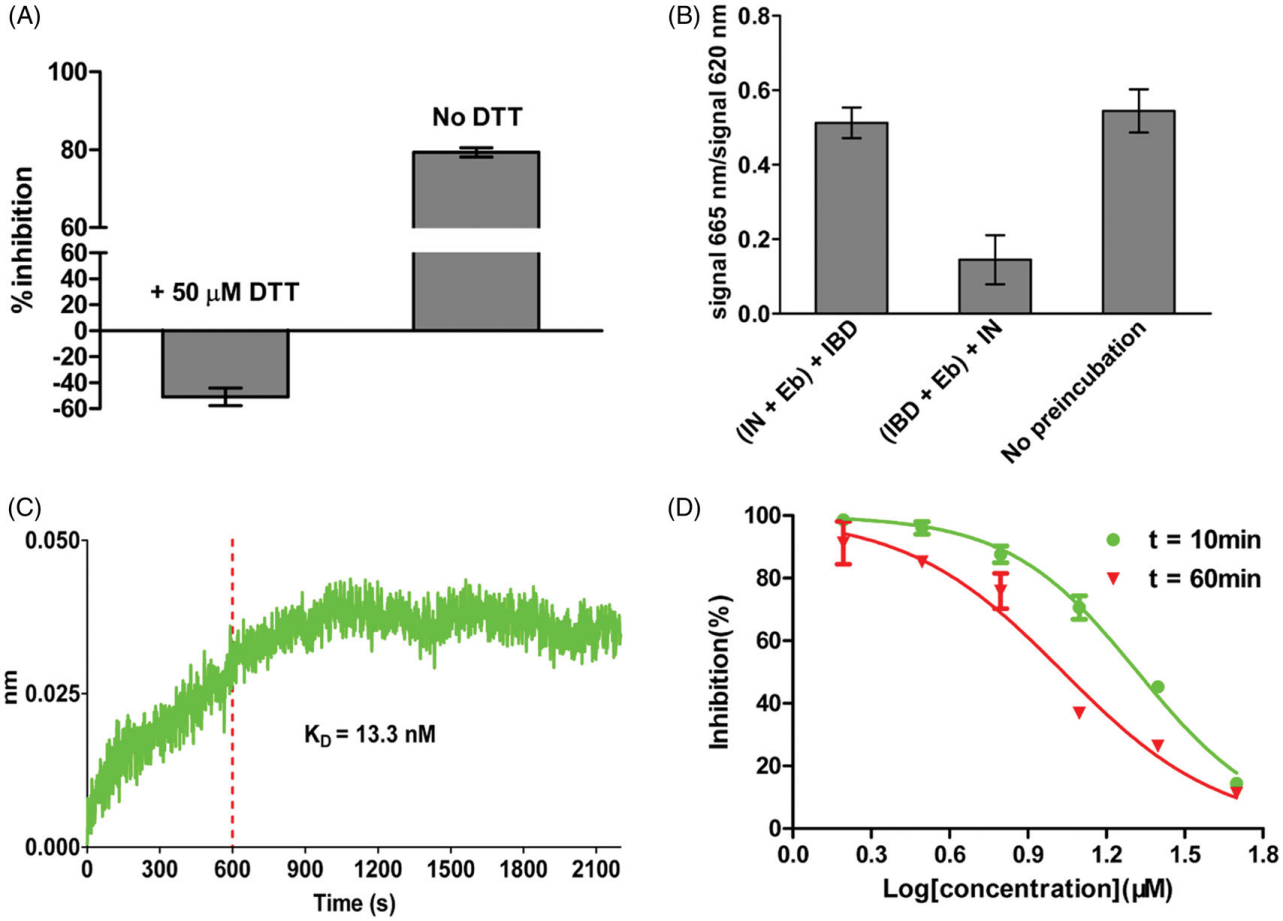
Determination of the inhibition mode of ebselen on LEDGF/p75-IN interaction. (A) The inhibition of ebselen on LEDGF/p75-IN interaction was abolished in the presence of 50 µM DTT. (B) Ebselen inhibited the LEDGF/p75-IN interaction by binding to LEDGF/p75. (C) Association/dissociation kinetics of ebselen for LEDGF/p75 determined by Octet. (D) Time-dependent inhibition of the LEDGF/p75-IN interaction with ebselen. The data are representative of results obtained in three independent experiments. Each point is carried out in triplicate; error bars show the mean ± SD.

Ebselen is a synthetic organoselenium compound, with anti-inflammatory, anti-oxidant and cytoprotective activity^33^. It is being investigated as possible treatments for reperfusion injury and stroke, hearing loss and tinnitus, and bipolar disorder^34^. Ebselen has also been investigated against infectious diseases (Table 3). Importantly, in most cases, ebselen was shown to be a covalent inhibitor of these previously studied proteins. Accordingly, we designed experiments to study the binding mode of ebselen. If ebselen functions by modifying reduced this in proteins, then adding compounds with reduced thiols should abrogate the ability of ebselen to inhibit the interaction. To test the hypothesis, we repeated the HTRF-based LEDGF/p75 IBD-IN interaction assay with ebselen in the presence of 50 µM dithiothreitol (DTT). The inhibitory effect of ebselen was completely abolished in the presence of DTT (Figure 4(A)). In a previous study, DTT was reported to inhibit LEDGF/p75-IN interaction (IC_50_ = 4.5 mM), but under the concentration (50 µM) used in our experiment, this inhibitory effect of DTT was negligible according to the precious research^35^. Then, we devised the following experiment to determine which protein (IN or LEDGF/p75) ebselen binds to. When IBD was pre-cubated with ebselen, there was a dramatical decrease in the HFRF signal, whereas no obvious signal change was observed when IN was pre-cubated with ebselen (Figure 4(B)). These results suggested that ebselen probably disrupt the interaction by binding to LEDGF/p75. Using biolayer interferometry (BLI) by Octet Red, we confirmed that ebselen binds to LEDGF/p75 with a K_D_ value of 13.3 nM (Figure 4(C)). If ebselen is a covalent inhibitor, it should show apparent increasing affinity over time^36^. The results showed that ebselen displayed an increase in its affinity over 1 h, consistent with covalent bond formation. (Figure 4(D)). All these data showed that ebselen probably inhibited the LEDGF/p75-IN interaction by covalently modifying the residues in LEDGF/p75.

**Table 3.** Activity of ebselen on targets from pathogen.

Target	IC_50_（µM）	Covalent	Reference
Cryptosporidium parvum glucose-6-phosphate isomerase	8.33	Yes	Eltahan et al.^46^
Tumor marker endothelial 8 and protective antigen interaction	1.7	Yes	Cryan et al.^37^
Trypanosoma brucei hexokinase 1	0.05	Yes	Gordhan et al.^47^
Escherichia coli thioredoxin reductase	0.52 (Ki)	Yes	Lu et al.^48^
C-terminal domain of HIV-1 capsid	0.047	Yes	Thenin-Houssier et al.^39^
Bacillus anthracis thioredoxin reductase	1	ND	Gustafsson et al.^49^
Clostridium difficile cysteine protease domain	0.0069	Yes	Bender et al.^50^
New Delhi metallo-β-lactamase	0.38	Yes	Chiou et al.^51^
Mycobacterium tuberculosis antigen 85	0.063	Yes	Favrot et al.^52^
Hepatitis C Virus NS3 Helicase-Nucleic Acid interaction	1.1/0.9	Yes	Mukherjee et al.^53^
Cryptosporidium parvum Inosine 5′-monophosphate dehydrogenase	0.71	Yes	Sarwono et al.^54^
Pseudomonas aeruginosa Diguanylate Cyclases	5	Yes	Lieberman et al.^55^

**Figure 5.**
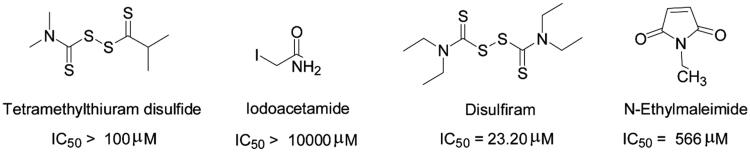
Ability of other thiol-modifying agents to inhibit LEDGF/p75 IBD-IN interaction.

However, ebselen seems to be a promiscuous compound. Data from PubChem reveals that ebselen was found to be active in 193 out of 1023 drug screenings, with 126 of these hits coming from confirmatory screens^37^. And the promiscuity of ebselen may be partly attributed to its known ability of modifying cysteine residues. If ebselen really bind LEDGF/p75 by modifying reduced thiols, then similar reagents should also bind LEDGF/p75 and inhibit the interaction. To test our hypothesis, we tested the sensitivity of LEDGF/p75-IN interaction to iodoacetamide, *N*-ethylmaleimide, disulphiram and tetramethylthiuram disulphide, four common reagents used to covalently modify deduced cysteines. Neither was a potent inhibitor of LEDGF/p75-IN interaction except for disulphiram (IC_50_ = 23.2 µM) (Figure 5). Obviously, ebselen is more potent PPI than other thiol-modifying compounds. Moreover, BLI experiment indicated that ebselen do not show obvious dissociation from LEDGF/p75 (Figure 4(C)). Taken together, as a highly active compound, ebselen probably formed selenium-sulphur bonds with reduced thiols in LEDGF/p75.

Development of LEDGF/p75-IN interaction inhibitors has been recognised as an attractive target for new antiviral drugs, and multiple studies have identified small-molecule inhibitors, all of which bind to IN. However, ligands binding to the LEDGF/p75 are more desired to avoid resistance resulting from mutation of the viral IN^38^. To the best of our knowledge, no potent small-molecule inhibitors directly binding to the IBD have been identified to date. In the present study, we showed for the first time that small-molecule compounds, ebselen inhibited LEDGF/p75-IN interaction by directly binding to LEDGF/p75. Although, a previous research indicated that ebselen inhibited replication of HIV-1 by targeting capsid^39^, the compound discovered here could be used as probe compounds to design and develop new inhibitors of LEDGF/p75-IN interaction. Moreover, LEDGF/p75 plays a critical role as an oncogenic cofactor of MLL fusion proteins^40^. LEDGF/p75 uses the same site to bind MLL as well as IN^41^. Therefore, the discovery of two LEDGF/p75-binding compounds will pave the way for development of novel drugs with dual applications for both M HIV and MLL leukaemias.

In summary, this study has identified a compound, ebselen, as novel LEDGF/p75-IN interaction inhibitor with new scaffolds via drug screening of three compound libraries. The research revealed that ebselen disrupted the interaction by covalently binding to LEDGF/p75. This study provides new clues for the development of LEDGF/p75-IN interaction inhibitors with novel scaffolds.
